# msBiodat analysis tool, big data analysis for high-throughput experiments

**DOI:** 10.1186/s13040-016-0104-6

**Published:** 2016-08-19

**Authors:** Pau M. Muñoz-Torres, Filip Rokć, Robert Belužic, Ivana Grbeša, Oliver Vugrek

**Affiliations:** Translational Medicine Group, Institut Rudjer Bošković, Division of Molecular Medicine, Bijenička Cesta 54, Zagreb, 10000 Croatia

**Keywords:** Bioinformatics, Data analysis, Proteomics, Data mining, Mass spectrometry, High-throughput analysis

## Abstract

**Background:**

Mass spectrometry (MS) are a group of a high-throughput techniques used to increase knowledge about biomolecules. They produce a large amount of data which is presented as a list of hundreds or thousands of proteins. Filtering those data efficiently is the first step for extracting biologically relevant information. The filtering may increase interest by merging previous data with the data obtained from public databases, resulting in an accurate list of proteins which meet the predetermined conditions.

**Results:**

In this article we present msBiodat Analysis Tool, a web-based application thought to approach proteomics to the big data analysis. With this tool, researchers can easily select the most relevant information from their MS experiments using an easy-to-use web interface. An interesting feature of msBiodat analysis tool is the possibility of selecting proteins by its annotation on Gene Ontology using its Gene Id, ensembl or UniProt codes.

**Conclusion:**

The msBiodat analysis tool is a web-based application that allows researchers with any programming experience to deal with efficient database querying advantages. Its versatility and user-friendly interface makes easy to perform fast and accurate data screening by using complex queries. Once the analysis is finished, the result is delivered by e-mail. msBiodat analysis tool is freely available at http://msbiodata.irb.hr

**Electronic supplementary material:**

The online version of this article (doi:10.1186/s13040-016-0104-6) contains supplementary material, which is available to authorized users.

## Background

High-performance techniques, such as mass spectrometry, are extremely powerful tools used in laboratories around the world [1, 2]. Those techniques are used to capture the existing proteins in an exact point of the cell cycle, and the output results in a list of thousand of proteins. Frequently, only a few of the records in the list are interesting for researchers proposes. To find those which want to be studied, an efficient filtering method is needed.

Different approaches have been proposed to this objective. By using a spreadsheet, results can be filtered according to the resulting score given in the experiment, but it does not provide any additional information of the proteins in the list. This limitation is overcome by PIQMIe [3] which also merges the data with proteomic information. The information resulting from PIQMIe analysis includes a concise graphical summary of the experiment, and a SQL file which can be downloaded. This file can be used to perform complex queries on the data in a relational database management system. However, it requires a previous knowledge on database querying. MaxQB Database [4] is a database focused on the quantitative analysis of proteomic data which compares the results of the experiment against others previously published. This comparison gives a clear idea of the changes occurred in the proteins concentrations of the sample due to a specific treatment, but it does not provide any type of qualitative information about the proteins in the sample. Among the commercial solutions that may help us to perform MS data interpretation, Ingenuity platform (IPA) from Qiagen [5] and ProteinCenter (https://www.thermofisher.com/order/catalog/product/IQLAAEGABSFALUMAZB) from Thermo Scientific are the most used. Meanwhile IPA covers different biological aspects of the data, from the genomic to its metabolomic activity, ProteinCenter is more specific for the proteomics analysis of the samples. Both services allow cross-evaluation of databases and thus simplifying the interpretation of results.

Those inconveniences can be overcome with msBiodat Analysis Tool. The service is intended to select relevant information from proteomics experiments, and can be especially useful to select proteins according to its GO [6] annotations. It provides an interesting information about protein localization, functions, or processes in which proteins are involved. The resulting information allows to identify easily which proteins can interact during a specific biological process.

## Implementation

### Web server implementation

msBiodat Analysis Tool is written in PERL 5.10.1-17, and it is hosted in a debian 6.0.10 server and runs Apache/2.2.16 web server. To manage spreadsheets, Spread-sheet::XLSX and Excel::Writer::XLSX libraries are used. Libraries used to manage mztab files were written by the authors of this work.

### Algorithm and decision work-flow

**Fig. 1:**
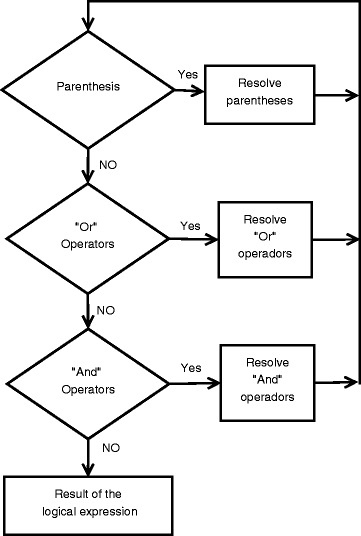
Scheme of the desission process. To evaluate the logical expressions, msBiodata follows the rules of precedence. First, the contend of the parenthesis is evaluated, then *and* and *or* connectors

The work-flow followed by the algorithm to select data depends on the used query. In general, each condition in the query is solved independently. Then the complete query is evaluated according the precedence of the connectors (*and* and *or*) and parenthesis (Fig. 1). If the evaluation of some clause depends on information not given at the spreadsheet, i.e. GO annotation, it is downloaded before resolving the clause.

## Results and discussion

A real data set will be used to show the possibilities of msBiodat. In this example, different options will be used to create complex query. To perform the example, the data from de Sousa Abreu et all [7], which can be found at http://www.marcottelab.org/MSdata/Data_01/, was downloaded from the MS/MS Shotgun Proteomics Data Repository (http://data.marcottelab.org/MSdata/). In the example is used the comparison cytosol-GFP versus MSI from the data set 1 (http://www.marcottelab.org/MSdata/Data_01/z_MSIGFP_CYTO.short.annot).

The objective of this study was to find an association between musashi1 down-stream targets and cancer disease in humans. The high-throughput techniques used in the study were: microarrays (RIP-Chip), the APEX pipeline [8, 9], and a MS-based proteomic technique. After performing the experiments, the proteins that were found to have different expression in both samples were manually classified according to their GO annotation. The classification can easily be automatized using msBiodata. In the following paragraphs an overview of how to perform the analysis is explained by the example above.

**Fig. 2:**
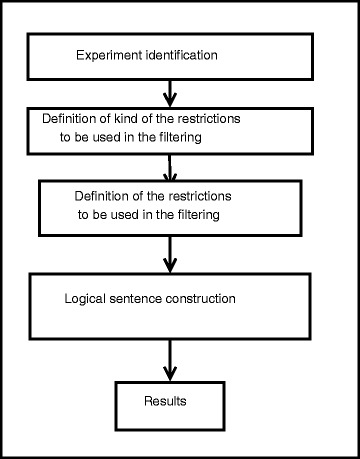
Schema of the four steps followed to enter the data. Four steps must be done before analyzing the data. Those are thought to identify and define the experiment, set up the comparisons to do, and join them in a logical expression. Finally, the data are analyzed, and the results delivered by mail to the researcher

The first steps of the data introduction are aimed to identify the experiment. It is necessary to fill in the following information: a project name, (to be included in the mail subject), an e-mail, and a file with the data to analyze. The file with the data can be formatted as an excel spreadsheet (XLSX) or a mzTAB file. It is also necessary to introduce where the data to be analyzed are placed. It can be a name of a sheet from an excel file, or molecule that want to be studied from a mzTAB file. In the following step, the type of statements to be used in the analysis are introduced. A statement is a condition that the selected data must meet. The web allows two types of statements which are thought to compare the content of the fields in the dataset against a value introduced by the user. Single statements are those where a unique field in the dataset is compared; it is also used for searching against GO database. On the other hand, double statements are used to evaluate the relationship between two fields of the same entry in the original data. In this example, one comparison of each type are performed. Last two steps before analyze the data are necessary to enter the statements to perform the data selection and to link them in a logical clause (Fig. 2).

The data set was downloaded as comma-separated value file (CSV) and converted to an XLSX file using a spreadsheet software (Additional file 1). The original CSV file had 27 rows of information that were removed, one row containing the header for each field in the document, and 2431 rows of data to analyze. In this case, the analysis is performed using two statements, one of each type, which are set-up by fulfilling the elements presented in the web (pop-up menus and text boxes). The single statement is aimed to identify putative proteins involved in cancer using the possibility of querying against GO. It requires both a protein identifier and the species to be studied (*Homo sapiens*) which are introduced at the boxes reserved to this propose. The protein identifiers can be the Ensembl [10] codes (ENSP and ENST), the UniProt [11] identification code, or the Gene codes. In the dataset, the protein codes are under the label #PROTEIN ID. The clause itself is introduced by using the label Gene ontology from the menu, the comparison symbol to be used is “equal to” (=), and the keyword *proliferation* which must be written in the text box. The box labeled as modifier can be used in those cases where is needed to discriminate fields with numeric values around 0. The second clause is aimed to find those proteins which have different expression in both samples. It can be done evaluating the difference of the APEX values of both samples by calculating its coefficient. The values around the threshold can be easily discriminate by applying a decimal logarithm to the coefficient. If the result is above 1, the evaluated protein is over-expressed at the cancer cells. As two fields from the original data are required, a double comparison must be used. The statement, *log2(APEX1/APEX2)>=1*, is introduced as it is explained above. This example also could be performed by using directly the field *APEX1/APEX2* from the data, nevertheless, using double comparison for a demonstration purpose is more appropriate. The last step is linking all together using logical connectors and parenthesis. The final query must look like *GeneOntology=proliferationandlog2(APEX1/APEX2)>=1*. The output resulting to this analysis will be a XLSX spreadsheet containing all the fields of the original data set with rows meeting the restrictions.

The versatility of the tool allows to increase the complexity of the queries in order to perform complex searches. An example can be a query aimed to select not only all over-expressed proteins involved in cell proliferation, but also those that are under-expressed and act as cell cycle suppressors. This query has two parts delimited by parenthesis and joined by an *or* operator which means that selected proteins must meet at least one of them. The tricky part for this query comes with the introduction of the GO terms. It is programmed to interpret all the terms written in the same text box as if they were linked by the *or* operator. To ensure that the selected proteins by the second part of the query will be involved in the cell cycle suppression, two GO statements must be included: *cycle*, and *suppression*. In this case, the final query is: *((Gene Ontology = proliferation and log2 (APEX 1/APEX 2)*>*= 1)****or****(Gene Ontology = cycle and Gene Ontology = suppression and log2 (APEX 1 /APEX 2)*<*1))*.

In the end, a special type of search will also be introduced: it will be used to find elements of a given field in a different one. This is performed by using *in* operator, as a way to compare the fields in a double clause. The result is that all items in the first field will also appear in the second field. In this case, no more elements are required.

## Conclusion

A tool to select data from the results of a mass spectrometry experiments is presented. The aim of the tool is to help researchers at the last steps of the high-throughput experiments by making data interpretation easy. It is thought as an easy-to-use and versatile web application which allows researchers to perform fast data selection by combining information from different sources. msBiodat Analysis Tool is especially useful to select proteins according its annotations, providing interesting information about processes where they are involved. Once the analysis is finished, the results are sent by e-mail to the researcher.

If a comparison against others tools is performed, the simplicity of msBiodat analysis tool presents some clear advantages. PIQMIe is the most similar tool to one presented in this article. It is thought as a data management, analysis and visualization tool, and returns both, a summary of the analyzed data, and an SQLite document. This document allows researchers to obtain interesting information by combining its data using a relational database management system. Nevertheless, querying those systems requires a previous knowledge. Our tool overcomes this inconvenient by implementing a guided path that researchers can follow to obtain the same information. ProteinCenter from thermo Scientific, and IPA ingenuity from Quiagen are powerful tools to investigate the relationship between the different proteins in a sample obtained from MS experiments. They allow to look inside putative pathways according the possible relations between proteins. To achieve this goal, the software query into different databases, and score each element in the sample according to the service criteria. As they are commercial products, a user license is required. In contrast, msBiodata is not able to build a pathway with the proteins in the sample, but it can be freely used by all the scientific community. MaxQB is a database for proteomics projects. It allows to compare projects and cells lines, and visualize the differences in the protein expression levels. It can be used to motorize the response of the cell in front of different treatments. Again, it is far from the objectives of msBiodata Analysis tool, but the combination of both tools can be interesting for researchers.

## Availability and requirements

Project name: msBiodatProject home page: http://msbiodata.irb.hrOperating system: LinuxProgramming language: PerlOther requirements: Perl + dependenciesLicense: GNU GPLAny restrictions to use by non-academics: noneThe code of the web page is available under request.

## Additional file

Additional file 1 Excel spreadsheets. XLSX file containing the data from Sousa Abreu et al. which is used in the example of the article. (XLSX 611 kb)
